# Phytolacca Decandra and Mint in Inflamed Mammæ

**Published:** 1884-05-20

**Authors:** 


**Phytolacca Decandra and Mint in Inflamed Mammæ.**— A writer in The Therapeutic Gazette says: Mint leaves steeped and applied to the mammæ will at once arrest the secretion of milk, and a most remarkable thing in connection with it, is that its action is strictly confined to the breast to which it is applied, checking the secretion in one and leaving the other to perform its natural function.

The poke-root fluid extract taken internally and applied locally, I hold as a specific for inflamed breasts, if used prior to the supurative stage. If administered early, it will in twelve hours begin to give relief, and in thirty-six hours all traces of inflammation will have subsided and disappeared.

